# Recent Progress on Heparin–Protamine Particles for Biomedical Application

**DOI:** 10.3390/polym14050932

**Published:** 2022-02-25

**Authors:** Yuuki Hata, Hiromi Miyazaki, Masayuki Ishihara, Shingo Nakamura

**Affiliations:** Division of Biomedical Engineering, National Defense Medical College Research Institute, 3-2 Namiki, Tokorozawa-shi 359-8513, Saitama, Japan; hata@ndmc.ac.jp (Y.H.); ishihara@ndmc.ac.jp (M.I.); snaka@ndmc.ac.jp (S.N.)

**Keywords:** molecular self-assembly, heparin, protamine, nanoparticle, microparticle, polyelectrolyte complex, biomedical application

## Abstract

Biomolecules are attractive building blocks with self-assembly ability, structural diversity, and excellent functionality for creating artificial materials. Heparin and protamine, a clinically relevant pair of biomolecules used in cardiac and vascular surgery, have been shown to coassemble into particulate polyelectrolyte complexes in vitro. The resulting heparin–protamine particles exhibit adhesive properties that enable advantageous interactions with proteins, cells, and various other substances and have been employed as functional materials for biomedical applications. In this review article, we summarize recent progress in research on the use of heparin–protamine particles as drug carriers, cell adhesives, and cell labels. Studies have demonstrated that heparin–protamine particles are potentially versatile in biomedical fields from drug delivery and regenerative medicine to plastic surgery.

## 1. Introduction

Biomolecules, such as proteins, nucleic acids, lipids and carbohydrates, form sophisticated assemblies via intermolecular interactions and constitute living organisms in nature. The self-assembly ability, structural diversity, and excellent functionality of biomolecules make them attractive as building blocks for creating artificial materials [1,2,3,4,5,6,7,8,9,10]. In fact, assembled biomolecular materials have found practical applications; clinical examples include collagen sponges used for hemostasis and wound healing [11,12] and lipid nanoparticles constituting drug products (e.g., COVID-19 vaccines) [13,14,15,16]. In addition to these clinically used materials composed of a single class of biomolecules, multicomponent assemblies have been explored to access new material properties and morphologies. For instance, the combination of proteins and nucleic acids is of interest in nanotechnology [17,18], while composites of lipid nanoparticles and polysaccharide hydrogels have been investigated as drug carriers [19]. These studies demonstrate that multicomponent biomolecular systems offer opportunities to generate a broad spectrum of functional materials.

Heparin and protamine are a clinically relevant pair of biomolecules. Heparin is a mixture of linear anionic polysaccharides with many sulfate groups and has been used clinically as an anticoagulant for more than 70 years [20,21,22,23]. In cardiac and vascular surgery, the use of the anticoagulant is followed by the administration of protamine, a small arginine-rich cationic protein, to neutralize the heparin [23,24,25,26]. The fact that pharmaceutical grade heparin and protamine are commercially available and clinically used in cardiac and vascular surgery makes these biomolecules attractive as building blocks for in vivo applications. The neutralization effect is a consequence of complex formation between cationic protamine and anionic heparin via electrostatic interactions and other intermolecular interactions. The complex formation in blood indicates that heparin and protamine can coassemble robustly even in crowded environments. Furthermore, the fact that the complexes form in patients’ blood suggests that heparin–protamine coassemblies have good biocompatibility, which encouraged us to explore this multicomponent system for the production of biomaterials.

**Figure 1:**
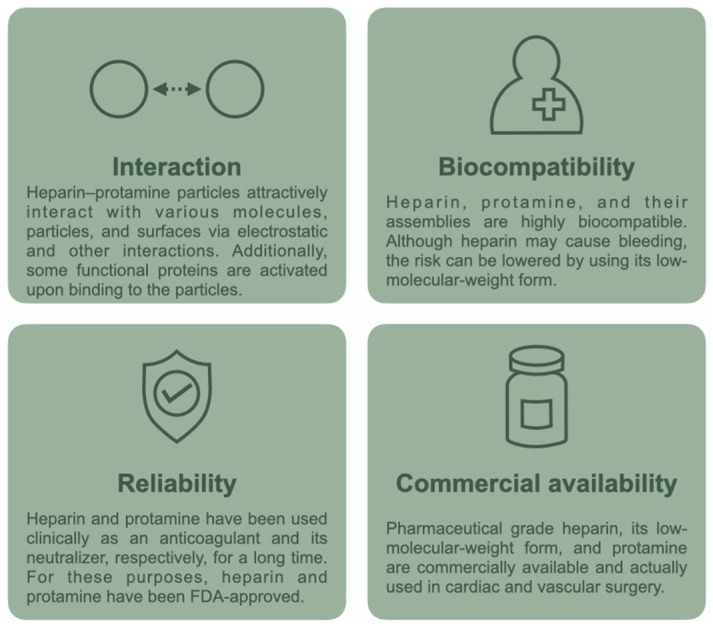
Attractive characteristics of the heparin–protamine system.

**Figure 2:**
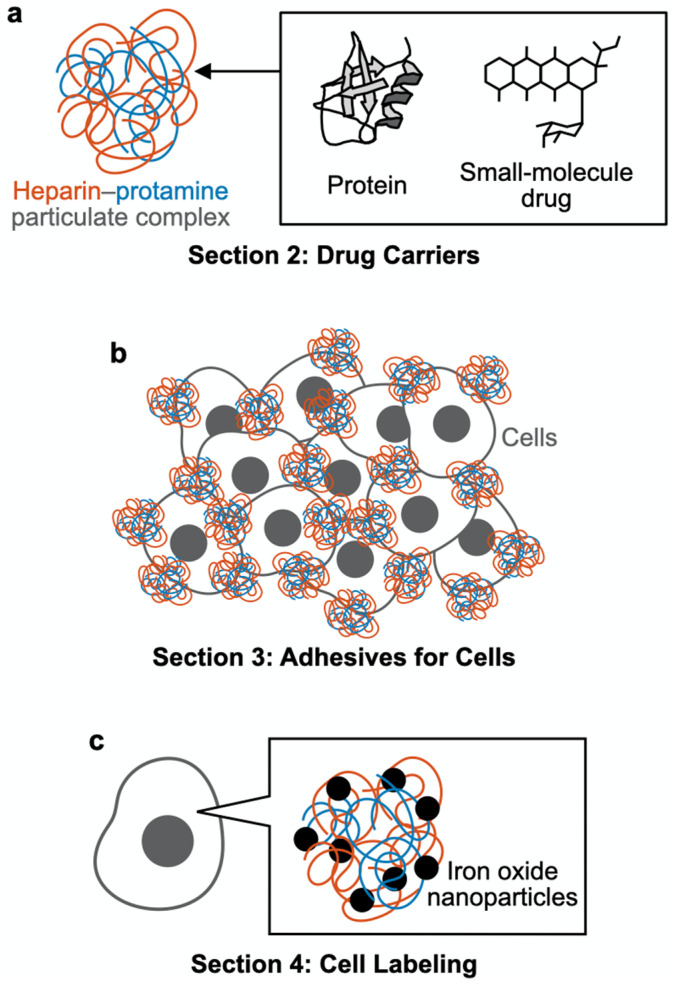
Schematic outline of this review article. (**a**) Section 2: Drug carriers. Heparin–protamine particles are useful as carriers for proteins and small-molecule drugs. (**b**) Section 3: Adhesives for cells. Heparin–protamine particles act as adhesives for cell aggregate formation. (**c**) Section 4: Cell labeling. Heparin–protamine complexes facilitate cell labeling with iron oxide nanoparticles.

Studies have explored the in vitro coassembly of heparin and protamine and demonstrated their coassembly into nanometer- or micrometer-sized particles that are useful for biomedical applications [27,28,29,30,31,32,33]. For particle preparation, low-molecular-weight heparin (several kDa), which is fractionated from heparin (10–20 kDa), is frequently used instead of heparin due to the lower risk of bleeding with the low-molecular-weight form [25,34,35]. In fact, we have developed particles from low-molecular-weight heparin as a drug delivery carrier with low bleeding risk for in vivo administration [28]. Heparin–protamine particles tend to have a net negative charge, as heparin is generally used in excess of protamine in terms of charge; a high fraction of protamine causes precipitation rather than particle formation [29]. Despite a net negative charge, polyelectrolyte complexes can exhibit attraction not only to positively charged substances but also to negatively charged proteins and surfaces through electrostatic interactions [36], partly due to the patchiness of charges on the protein surface and the charge regulation for proteins by counterpart polyelectrolytes [37]. In addition, the particles exhibit strong interactions with heparin-binding proteins; notably, the bioactivities of such proteins tend to be enhanced upon binding to heparin. These characteristics, coupled with the biocompatibility and commercial availability of heparin and protamine (Figure 1), have led to the use of particulate heparin–protamine complexes as carriers for proteins [28,29,38,39,40,41,42,43,44,45] and adhesives for cells [46,47,48]. Such studies demonstrate that heparin–protamine particles show great promise as versatile nanomaterials in biomedical engineering and medicine. In this review article, we summarize recent research progress on heparin–protamine particles for biomedical applications (Figure 2).

## 2. Drug Carriers

As stated above, heparin–protamine particles are useful as carriers for proteins due to their ability to adsorb proteins via electrostatic interactions and other intermolecular interactions. The particles can preserve loaded proteins from degradation by protease and from heat inactivation [29]. Moreover, loaded proteins are released from the particles in a controlled manner, probably through enzymatic degradation of heparin and protamine. A study reported that the subcutaneously injected heparin–protamine particles disappeared visually after 14 d [28]. This section introduces recent studies that further investigated heparin–protamine particles as drug carriers for clinical application. Notably, heparin–protamine particles have sometimes been subjected to chemical and nonchemical modification to develop advanced drug carriers.

### 2.1. Intact (Nonmodified) Particles

The potential of heparin–protamine particles as drug carriers has been investigated, especially for fibroblast growth factor (FGF)-2, a protein that stimulates cell proliferation and is used clinically in wound care [49,50,51]. FGF-2 has the advantageous ability to strongly bind to heparin and heparin-like molecules and, moreover, is activated by its binding to heparin [20,21,52,53]. These characteristics have prompted us to use heparin–protamine particles as carriers of FGF-2 for various applications.

FGF-2-containing heparin–protamine nanoparticles were used for the treatment of crush syndrome [54]. A rat model of crush syndrome was prepared by compressing the hind limbs of anesthetized rats using a device, followed by the local administration of FGF-2-containing heparin–protamine nanoparticles. The treated rats exhibited a higher score in motor function, better blood flow, a higher number of blood vessels, and faster recovery of muscle tissue than rats administered FGF-2 alone (i.e., without heparin and protamine). Another study investigated the potential of heparin–protamine carriers for wound care associated with radiation therapy [55]; although radiation therapy is effective for cancer treatment, radiation exposure tends to cause a delay in wound healing as a side effect. Cutaneous full-thickness defect wounds in the backs of rats were made with a punch and a sharp blade. Although X-ray irradiation delayed wound healing, FGF-2-containing heparin–protamine nanoparticle administration prior to irradiation led to a significantly shorter delay accompanied by vascularization, fibrous tissue formation, and fewer apoptotic dermal fibroblasts. Studies on crush syndrome and irradiated wounds demonstrated that FGF-2-containing heparin–protamine particles can promote the healing of various kinds of injury.

**Figure 3:**
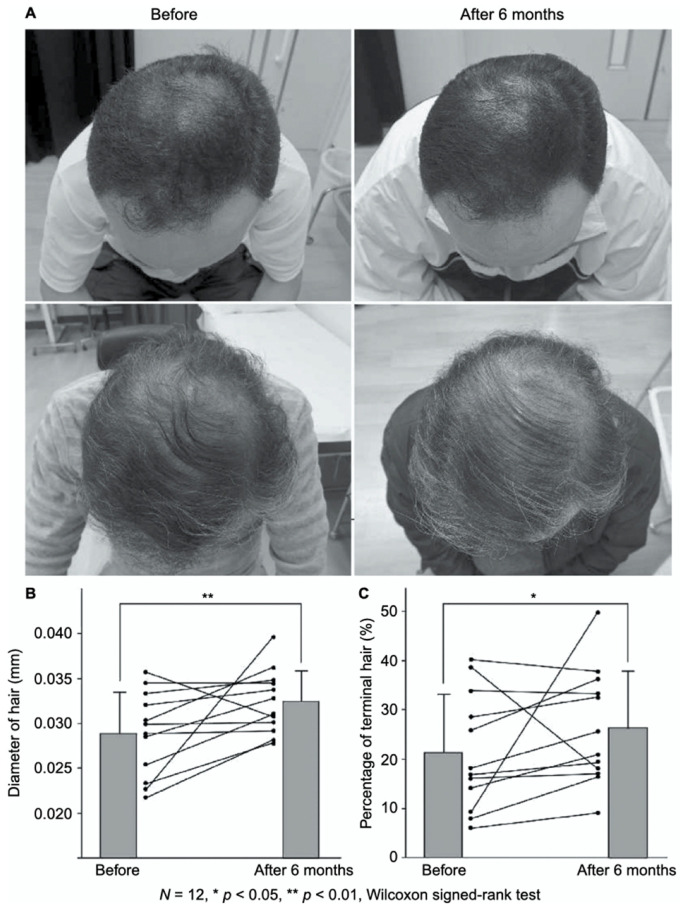
Promotion of hair growth by FGF-2-containing heparin–protamine nanoparticles. (**A**) Representative photographs of improved cases after 6 months of treatment. Increases in (**B**) the hair diameter and (**C**) the percentage of terminal hair. Adapted from Ref. [56].

FGF-2-containing heparin–protamine nanoparticles were shown to promote hair growth in a clinical study (Figure 3) [56]. Twelve participants with thin hair transdermally applied FGF-2-containing nanoparticle dispersions to the skin of their scalps twice a day for 6 months, resulting in an increase in the mean diameter of their hairs. Objective improvements in thin hair were observed in two cases. Additionally, nine participants experienced greater bounce and hair resilience. Thus, the transdermal application of FGF-2-containing heparin–protamine nanoparticles to the scalp has potential as a new treatment for alopecia.

Heparin–protamine particles can carry not only FGF-2 but also other proteins. In fact, various growth factors contained in platelet-rich plasma were loaded into heparin–protamine particles [38]. Notably, many growth factors in platelet-rich plasma exhibit heparin-binding ability and are activated upon binding, similar to FGF-2. The resultant complexes containing growth factors from platelet-rich plasma were administered to split-thickness skin graft donor site wounds in male rats [57]. The treatment effectively promoted epithelialization and new vessel formation, suggesting that platelet-rich plasma-containing heparin–protamine particles are useful in healing split-thickness skin wounds.

### 2.2. Nonchemically Modified Particles

The nonchemical modification of heparin–protamine particles has been demonstrated to be a promising strategy for creating advanced drug carriers. Nonchemical modification strategies are generally advantageous in terms of simplicity compared with the chemical modification strategies described in the next subsection. Additionally, the use of US Food and Drug Administration (FDA)-approved and clinically used drugs (i.e., intact heparin and protamine) may contribute to shortening the time required for safety evaluation, even for off-label use.

**Figure 4:**
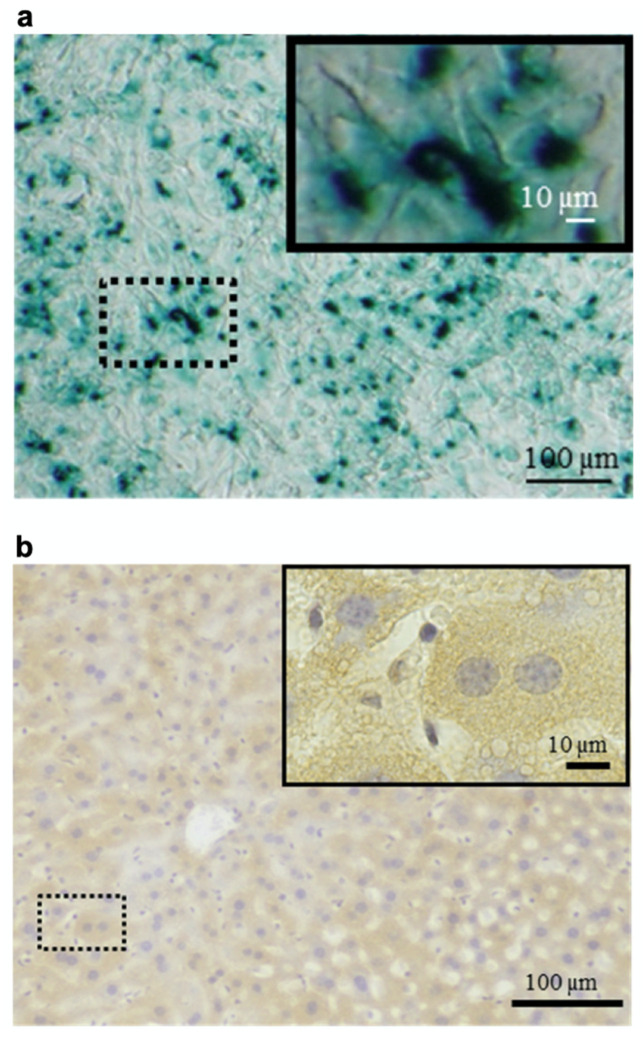
Protein delivery using heparin–protamine nanoparticles decorated with a cell-penetrating peptide. (**a**) In vitro delivery of β-galactosidase into cells. Blue cytoplasmic deposits indicate successful delivery. (**b**) In vivo delivery of β-galactosidase to mouse hepatocytes through hydrodynamics-based injection. Brown cytoplasmic deposits observed throughout the liver specimens indicate successful delivery. Adapted from Ref. [36].

Heparin–protamine complexes tend to have a net negative charge because anionic heparin is the major component, as stated above. The net negative charge was exploited for the nonchemical modification of particles through electrostatic interaction with a cationic peptide, GRKKRRQRRRPPQ (Figure 4) [36]. This sequence is derived from the human immunodeficiency virus-1 (HIV-1) viral protein TAT (trans-activator of transcription) and is known as a cell-penetrating peptide [58,59]. Thus, the cationic peptide was used to endow heparin–protamine nanoparticles with transmembrane transport ability. The cationic peptide was adsorbed onto heparin–protamine nanoparticles, making the net charge of the particles less negative. The resultant peptide-decorated nanoparticles were loaded with model proteins, namely, β-galactosidase and RNase T1. It is noted that both of the loaded proteins have net negative charges, suggesting that those proteins interacted with the polyelectrolyte complexes through local charges in proteins. In vitro experiments revealed that the peptide-decorated nanoparticles transported proteins into cells due to the cell-penetrating ability derived from the attached peptides. Furthermore, targeted protein delivery to mouse hepatocytes was achieved in vivo with peptide-decorated nanoparticles through a hydrodynamics-based injection method.

While proteins can be loaded easily into heparin–protamine particles as described above, small-molecule drug-loading efficiency tends to be relatively low. To improve the efficiency, calcium carbonate (CaCO_3_) was incorporated into heparin–protamine complexes [60]. The organic–inorganic hybrid drug carriers were prepared by mixing the solution containing protamine and CO_3_^2−^ and the solution containing heparin and Ca^2+^ under particular conditions, resulting in particles with a vesicular morphology. The presence of CaCO_3_ increased the loading efficiency of a small-molecule drug, doxorubicin, possibly due to the presence of nanopores in the inorganic–polymer hybrid assemblies and decreased drug permeability by CaCO_3_. Another small-molecule drug, tariquidar, was also loaded at a low content into the hybrid nanovesicles. In addition to increased drug loading capacity, CaCO_3_, which has a relatively high water solubility at a low pH, endowed the system with pH sensitivity; the loaded antitumor drugs were preferentially released at lower pH. This pH sensitivity was favorable for drug delivery to tumor sites with a relatively low pH. In vitro experiments with nonresistant cells (HeLa and MCF-7) and drug-resistant cancer cells (MCF-7/ADR) showed that the dual drug-loaded nanovesicles exhibited improved tumor cell inhibitory efficiency, especially for drug-resistant cells. In a later study, a tumor-targeting ligand, biotin, was additionally introduced into hybrid nanovesicles to enhance cell uptake through biotin receptor-mediated endocytosis [61].

### 2.3. Chemically Modified Particles

Chemical modification is a powerful strategy to generate various functional drug carriers from heparin and protamine. To date, controlled release of small-molecule drugs, oral delivery, and improved anticancer efficacy have been achieved by using chemically modified heparin–protamine particles, as shown below.

Heparin–protamine nanocapsules were chemically crosslinked to serve as carriers of small-molecule drugs [62]. The nanocapsules were prepared by the layer-by-layer assembly of heparin and protamine on a silica template, followed by loading of the anticancer drug doxorubicin and chemical crosslinking. Chemical crosslinking prevented the premature release of loaded doxorubicin, possibly due to decreased permeability of the nanocapsule walls. In vitro experiments using MCF-7 breast cancer cells showed that the nanocapsules were readily internalized and degraded inside the cells, releasing the loaded doxorubicin and causing cancer cell death.

Bile acid-conjugated heparin–protamine nanoparticles were found to be orally available [63]; oral availability is a challenging characteristic for biomolecular nanoparticles due to biological barriers in the body [64,65,66]. After chemical conjugation with bile acid, low-molecular-weight heparin was mixed with protamine for nanoparticulate complex formation [63]. The bile acid-conjugated nanoparticles successfully attached to the enterocyte surface and were then internalized by the cells through interaction between the bile acid on the nanoparticles and the bile acid transporters of the cells. Animal experiments using nude mice revealed that orally administered nanoparticles interacted with bile acid transporters in the ileum and were taken up by epithelial cells.

For antiangiogenic therapy, a low-molecular-weight heparin–taurocholate conjugate—LHT7—which contains ~7 taurocholate groups in a heparin chain, has been developed and shown to act as an angiogenesis inhibitor [67,68,69]. Nevertheless, LHT7 showed toxicological effects including liver functional disturbances and limited anticancer effects [69]. To increase therapeutic duration while decreasing liver toxicity, PEGylated LHT7 was assembled with protamine to form nanoparticulate complexes [70]. The LHT7-containing nanoparticles exhibited improved antiangiogenic effects through the extended circulation and tumor accumulation of nanoparticles and the continued slow release of PEGylated LHT7. Notably, the nanoparticles diffused through leaky tumor blood vessels and extravasated through the blood vessels surrounding the collagen layer. A later study performed PEGylation on protamine, rather than heparin derivatives, to prevent undesirable structural changes to the heparin derivatives by the PEGylation process [71].

More recently, PEGylated LHT7–protamine nanoparticles were used as carriers for tumor necrosis factor-related apoptosis-inducing ligand (TRAIL) [72], which exhibits selective cytotoxicity to cancer cells for cancer therapeutics but has low stability and a short half-life [73,74,75]. It was found that the loading of TRAIL into the nanoparticles improved both the pharmacokinetic properties and the tumor accumulation rate while maintaining the tumor-selective cytotoxicity of TRAIL [72]. Histological analysis revealed both antiangiogenic efficacy and the homogeneous induction of cancer cell apoptosis by the PEGylated LHT7–TRAIL–protamine nanocomplexes, suggesting synergistic antitumor effects of accumulated TRAIL and LHT7 in tumor tissue.

## 3. Adhesives for Cells

Heparin–protamine particles have attractive interactions with cells as well as proteins and, consequently, have been investigated as adhesives for cells. Recent studies demonstrated the application of heparin–protamine adhesives to cell culture, cell transplantation, and skin grafting.

### 3.1. Cell Culture

Heparin–protamine nanoparticles were used as coating materials for cell culture plastic plates to enhance the adhesion and growth of cells [76]. When the nanoparticle dispersions were applied to cell culture plates, the nanoparticles were adsorbed onto the plastic surfaces to form a stable coating layer. Adipose-derived stromal cells and bone marrow-derived mesenchymal stem cells adhered well to the coated plates due to the adhesive properties of the heparin–protamine layer. Moreover, the heparin–protamine coating layers seemed to adsorb various heparin-binding substances from platelet-rich plasma supplemented with FGF-2, stimulating cell proliferation. Importantly, these cells maintained their multilineage potential for differentiation into adipocytes or osteoblasts. A later study demonstrated the three-dimensional culture of various human cells by using human plasma–medium gels containing heparin–protamine microparticles [77].

### 3.2. Cell Transplantation

Cell transplantation is a promising therapeutic strategy for tissue regeneration [78,79,80]. Nevertheless, there are still challenges, including poor survival and integration of transplanted cells in the targeted tissues. For cell transplantation, heparin–protamine microparticles were used as adhesives for the production of cell aggregates [81]. Human synovial mesenchymal stem cells formed aggregates upon mixing with the adhesive microparticles while maintaining cell viability. When injected into a cartilage defect model in the pig femoral trochlea, cell aggregates with heparin–protamine microparticles were prevented from leaking from the transplanted site. Additionally, further experiments using an osteoarthritic rabbit model suggested that the cell aggregates regenerated cartilage defects even in patients with advanced osteoarthritis, although the mechanisms mediating regeneration of cartilage and cardiomyocytes have yet to be elucidated.

**Figure 5:**
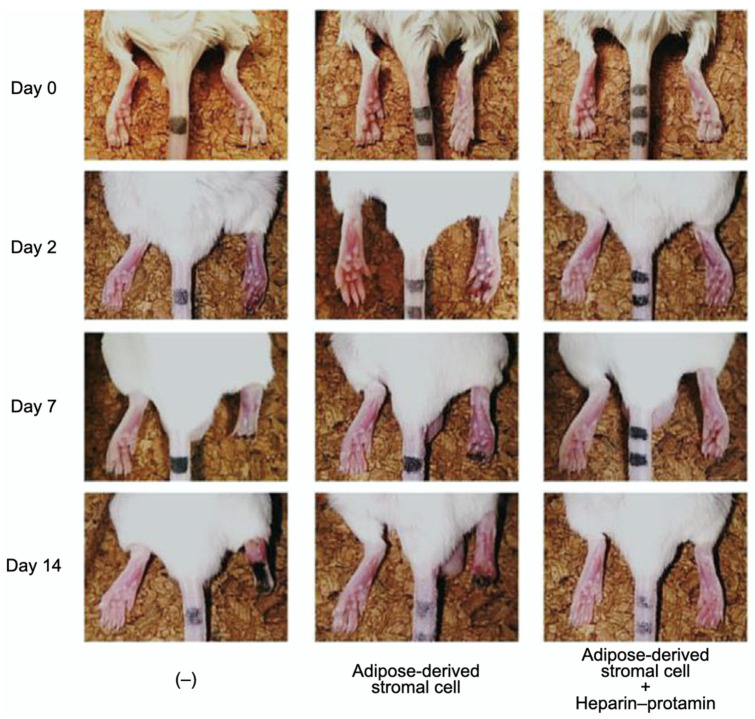
Amelioration of limb ischemia in a mouse model by the transplantation of adipose-derived stromal cell aggregates with heparin–protamine particles. Adapted from Ref. [82].

Adipose-derived stromal cell aggregates with heparin–protamine particles were shown to ameliorate limb ischemia in a mouse model (Figure 5) [82]. The cell aggregates were allotransplanted into unilateral hindlimb ischemic muscles induced in adult mice by ligation of the iliac artery and hindlimb vein. Cell transplantation promoted neovascularization and prevented ischemic limb loss. Heparin–protamine particles seemed not only to induce cell aggregate formation but also to immobilize, retain, and gradually release various heparin-binding growth factors from adipose-derived stromal cells, leading to sustained vascularization.

### 3.3. Skin Grafting

Skin grafting is a common technique for treating burns, chronic ulcers, and skin defects after cutaneous surgical procedures [83,84,85]. Nevertheless, skin grafts tend to suffer from stagnated revascularization, which leads to poor outcomes. It was reported that heparin–protamine particles were useful to increase the survival rate of full-thickness skin grafts [86]. Heparin–protamine particles and various growth factors from platelet-rich plasma were injected into full-thickness skin wounds created on the dorsal skin of rats, followed by full-thickness skin grafting. This therapeutic approach effectively promoted the survival rate of full-thickness skin grafts with increased blood flow and new vessel formation at the grafting site.

## 4. Cell Labeling

Heparin–protamine complexes were reported to facilitate cell labeling with iron oxide nanoparticles as described below. This cell labeling method found applications in the magnetic resonance imaging (MRI) and magnetic targeting of transplanted cells.

### 4.1. MRI

**Figure 6:**
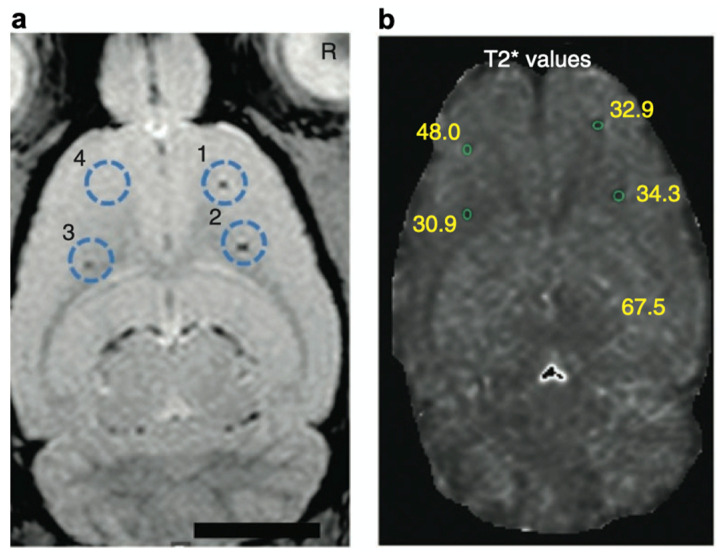
In vivo magnetic resonance visualization of intracranially implanted cells labeled with heparin–protamine–ferumoxytol complexes. (**a**) A magnetic resonance image. Circles indicate injection sites of the labeled cells. (**b**) Calculated T2* map with T2* values at each injection site. Adapted by permission from Nature Publishing Group: Springer Nature Ref. [91], Copyright 2012.

Cell-based therapies have attracted considerable attention in regenerative medicine [87,88]. To understand the therapeutic effects, noninvasive imaging approaches have been developed for monitoring the migration of cell products [89,90]. In this context, heparin and protamine were employed to label cells with ferumoxytol, a superparamagnetic iron oxide nanoparticle, for in vivo MRI (Figure 6) [91]. It should be highlighted that all of the components—namely, heparin, protamine, and ferumoxytol—are FDA-approved drugs, although this use is off label. As a cell labeling experiment, heparin, protamine and ferumoxytol were added to hematopoietic stem cells, neural stem cells, bone marrow stromal cells, and T cells. As a result, ternary nanocomplexes composed of heparin, protamine, and ferumoxytol were internalized into the endosomes of those cells. No long-term effect or toxicity on cellular physiology or function was observed for the cells labeled with the ternary nanocomplexes. In vivo MRI successfully visualized labeled human bone marrow stromal cells that had been intracranially implanted in rat brains.

After pioneering work, fundamental and preclinical studies have been conducted on the clinical use of the heparin–protamine–ferumoxytol cell labeling system for MRI [92,93,94,95]. A preclinical study optimized the labeling protocol and investigated the efficacy for MRI and the safety/toxicity of this cell labeling system [92]. Neural stem cells labeled through the optimized protocol were viable and proliferative and retained their tumor tropism in vitro. MRI revealed the dynamic in vivo distribution of the labeled cells after intracerebral or intravenous injection into glioma-bearing mice. Preclinical studies of the labeled cells intracerebrally administered to mice showed no significant clinical or behavioral changes, no neuronal or systemic toxicities, and no abnormal accumulation of iron in the liver or spleen. This report has led to a clinical trial of the heparin–protamine–ferumoxytol cell labeling system for posttransplant MRI visualization and tracking.

### 4.2. Magnetic Targeting

Stem cell transplantation is a promising therapeutic strategy for acute or chronic ischemic cardiomyopathy [96]. Nevertheless, its efficacy tends to suffer from the low efficiency of cell retention and engraftment, partly due to the “wash-out” of cells by coronary blood flow and heart contraction [97]. To overcome this issue, the magnetically targeted delivery of cells labeled with heparin–protamine–ferumoxytol complexes was investigated [98]. Rat cardiosphere-derived stem cells were labeled with ternary complexes and intracoronarily infused into syngeneic rats. Magnetic targeting successfully increased the cardiac retention of the transplanted cells without cardiac inflammation and iron overload, leading to attenuated left ventricular remodeling and therapeutic benefit.

## 5. Summary and Outlook

This review summarizes recent research progress on heparin–protamine particles for drug carriers, cell adhesives, and cell labels (Table 1). One of the most important characteristics of the biomolecular polyelectrolyte complex is the adhesive property, which is manifested via electrostatic interactions and other intermolecular interactions. This characteristic allows the loading of various substances, such as proteins and nanoparticles, and the adhesion of cells. Consequently, heparin–protamine particles are potentially versatile in various biomedical fields from drug delivery and regenerative medicine to plastic surgery. The fact that both components are commercially available as pharmaceuticals and are clinically used in surgery suggests that this multicomponent biomolecular system shows great promise for practical applications. In fact, some applications introduced in this review are under clinical or preclinical investigation.

Despite recent progress, there is still plenty of room for further research into heparin–protamine particles. Their fundamental characteristics, including their internal structure, interaction with proteins and other substances, physicochemical and biological stability, and pharmacokinetics, have yet to be fully revealed. Furthermore, the modification of heparin–protamine particles is still in the early stages of research. We are especially interested in nonchemical modification with poly- and oligoelectrolytes, which offer a large variety of functionalities and can be readily incorporated into polyelectrolyte complexes via electrostatic adsorption. Attractive candidates include synthetic aptamers [99] and peptide growth factors [100], which will endow heparin–protamine complexes with excellent biofunctionalities. The resulting functionalized particles will find novel biomedical applications. Further safety testing is necessary for heparin–protamine particles; although heparin and protamine are FDA-approved drugs in clinical use, the biomedical applications described in this review are off-label uses. Nevertheless, the fact that both components have been FDA-approved for some purposes will contribute to shortening the time required for safety evaluation of the particles.

It appears that heparin–protamine particles have been developed mostly in biomedical engineering and medicine. Nevertheless, further investigations from the perspective of molecular self-assembly may lead to innovations in assembled heparin–protamine materials. It is hoped that this review will inspire polymer and materials scientists to contribute to developing advanced biomedical materials via heparin–protamine coassembly.

## Figures and Tables

**Table 1:** Recent studies on the biomedical application of heparin–protamine particles.

Section	Application	Additive/Modification for Particles	Ref.
2. Drug carriers	Healing of crush syndrome with FGF-2	-	[54]
	Healing of irradiated wounds with FGF-2	-	[55]
	Healing of skin graft donor sites with platelet-rich plasma	-	[57]
	Hair growth with FGF-2	-	[56]
	Targeted protein delivery to mouse hepatocytes	Cell-penetrating peptide	[36]
	Antitumor drug delivery in vitro	CaCO_3_	[60]
	Targeted antitumor drug delivery in vitro	CaCO_3_, conjugation of biotin	[61]
	Antitumor drug delivery in vitro	Chemical crosslinking	[62]
	Oral delivery	Conjugation of bile acid	[63]
	Antiangiogenic therapy of tumors	Conjugation of taurocholate, PEGylation	[70]
	Antiangiogenic therapy of tumors	Conjugation of suramin, PEGylation	[71]
	Proapoptotic and antiangiogenic therapy of tumors with TRAIL	Conjugation of taurocholate, PEGylation	[72]
3. Adhesives for cells	Two-dimensional cell culture	-	[76]
	Three-dimensional cell culture	-	[77]
	Cell transplantation for cartilage regeneration	-	[81]
	Cell transplantation for ameliorating limb ischemia	-	[82]
	Improving the survival of full-thickness skin grafts with platelet-rich plasma	-	[86]
4. Cell labeling	Cell tracking by MRI	Ferumoxytol (iron oxide nanoparticles)	[91,92,93,94,95]
	Magnetically targeted delivery of cells	Ferumoxytol (iron oxide nanoparticles)	[98]
